# Pregnancy vitamin D supplementation and offspring bone mineral density in childhood follow-up of a randomized controlled trial

**DOI:** 10.1016/j.ajcnut.2024.09.014

**Published:** 2024-09-19

**Authors:** Rebecca J Moon, Stefania D’ Angelo, Elizabeth M Curtis, Kate A Ward, Sarah R Crozier, Inez Schoenmakers, M Kassim Javaid, Nicholas J Bishop, Keith M Godfrey, Cyrus Cooper, Nicholas C Harvey, the MAVIDOS Trial Group, Elaine M Dennison, Richard Eastell, Robert Fraser, Saurabh V Gandhi, Hazel M Inskip, Stephen H Kennedy, Aris T Papageorghiou, Ann Prentice

**Affiliations:** 1 MRC Lifecourse Epidemiology Centre, University of Southampton, Southampton General Hospital, Tremona Road, Southampton, United Kingdom; 2 Paediatric Endocrinology, Southampton Children’s Hospital, University Hospital Southampton NHS Foundation Trust, Southampton General Hospital, Tremona Road, Southampton, United Kingdom; 3 MRC Versus Arthritis Centre for Musculoskeletal Health and Work, MRC Lifecourse Epidemiology Centre, University of Southampton, Southampton, United Kingdom; 4 NIHR Applied Research Collaboration Wessex, Southampton Science Park, Innovation Centre, 2 Venture Road, Chilworth, Southampton, United Kingdom; 5 NIHR Southampton Biomedical Research Centre, University of Southampton and University Hospital Southampton NHS Foundation Trust, Southampton General Hospital, Tremona Road, Southampton, United Kingdom; 6 Faculty of Medicine and Health Sciences, University of East Anglia, Norwich, United Kingdom; 7 NIHR Biomedical Research Centre, University of Oxford, United Kingdom; 8 Nuffield Department of Orthopaedics, Rheumatology and Musculoskeletal Sciences, University of Oxford, Oxford, United Kingdom; 9 Division of Clinical Medicine, School of Medicine and Population Health, University of Sheffield, Sheffield, United Kingdom

**Keywords:** bone mineral density, cholecalciferol, developmental programming, pregnancy, randomized controlled trial, vitamin D

## Abstract

**Background:**

Findings from the Maternal Vitamin D Osteoporosis Study (MAVIDOS) trial demonstrated a positive effect of gestational cholecalciferol supplementation on offspring bone mineral density (BMD) at age 4 y. Demonstrating the persistence of this effect is important to understanding whether maternal vitamin D supplementation could be a useful public health strategy to improving bone health.

**Objectives:**

We investigated whether gestational vitamin D supplementation increases offspring BMD at ages 6–7 y in an exploratory post-hoc analysis of an existing trial.

**Methods:**

In the MAVIDOS randomized controlled trial, pregnant females <14 wk’ gestation with a singleton pregnancy and serum 25-hydroxyvitamin D 25–100nmol/l at 3 United Kingdom hospitals (Southampton, Sheffield, and Oxford) were randomly assigned to either 1000 IU/d cholecalciferol or placebo from 14 to 17-wk gestation until delivery. Offspring born at term to participants recruited in Southampton were invited to the childhood follow-up at ages 4 and 6–7 y. The children had a dual-energy X-ray absorptiometry (DXA, Hologic discovery) scan of whole-body-less-head (WBLH) and lumbar spine, from which bone area, bone mineral content (BMC), BMD, and bone mineral apparent density (BMAD) were derived. Linear regression was used to compare the 2 groups adjusting for age, sex, height, weight, duration of consumption of human milk, and vitamin D use at 6–7 y.

**Results:**

A total of 454 children were followed up at ages 6–7 y, of whom 447 had a usable DXA scan. Gestational cholecalciferol supplementation resulted in higher WBLH BMC [0.15 SD, 95% confidence interval (CI): 0.04, 0.26], BMD (0.18 SD, 95% CI: 0.06, 0.31), BMAD (0.18 SD, 95% CI: 0.04, 0.32), and lean mass (0.09 SD, 95% CI: 0.00, 0.17) compared with placebo. The effect of pregnancy cholecalciferol on bone outcomes was similar at ages 4 and 6–7 y.

**Conclusions:**

Supplementation with cholecalciferol 1000 IU/d during pregnancy resulted in greater offspring BMD and lean mass in mid-childhood compared with placebo in this exploratory post-hoc analysis. These findings suggest that pregnancy vitamin D supplementation may be an important population health strategy to improve bone health.

**Trial registration number:**

This trial was registered at the ISRCTN (https://doi.org/10.1186/ISRCTN82927713) as 82927713 and EUDRACT (https://www.clinicaltrialsregister.eu/ctr-search/trial/2007-001716-23/results) as 2007-001716-23.

## Introduction

Vitamin D has a recognized role in calcium homeostasis and skeletal health. There is increasing evidence that also suggests the importance of vitamin D to skeletal development during fetal and early postnatal life [1,2]. In observational studies, maternal 25-hydroxyvitamin D [25(OH)D] status has been positively associated with offspring bone mineral density (BMD) and/or bone mineral content (BMC) at birth [3,4], during childhood [5,6], and at peak bone mass [7], although these findings are not consistent across all cohorts [1,[8], [9], [10]].

Results from intervention studies also suggest beneficial effects of gestational vitamin D supplementation on offspring BMD in early childhood [2]. In the MAVIDOS randomized placebo-controlled trial of pregnancy vitamin D supplementation in the United Kingdom [11], we demonstrated a positive effect of 1000 IU/d cholecalciferol during pregnancy on offspring whole-body-less-head (WBLH) BMD at age 4 y [12]. Interestingly, there was no difference in offspring whole body BMC or BMD at birth between the 2 groups [13]. This complemented the findings of the Copenhagen Prospective Studies on Asthma in Childhood (COPSAC_2010_) trial in Demark, in which high-dose maternal vitamin D supplementation (2800 IU/d) increased offspring whole body BMC and BMD at age 6 y compared with low-dose supplementation (400 IU/d), with similar but weaker effects at age 3 y in a subset of children [14]. Together, these findings suggest that an effect of gestational vitamin D supplementation on the offspring skeleton might evolve over childhood [2]. This is supported by a study in a small subset of children born into the MAVIDOS study that showed greater bone anabolic response to stimulation in those born to mothers randomly assigned to vitamin D supplementation [15]. We therefore sought to establish the persistence and/or evolution of the effect of gestational vitamin D supplementation on offspring BMD at 6–7 y in the MAVIDOS trial.

## Methods

MAVIDOS was a double-blind randomized placebo-controlled trial of gestational vitamin D supplementation [11]. The trial and subsequent follow-up phases were approved by the Southampton and South-West Hampshire Research Ethics Committee and registered prospectively (ISRCTN:82927713; EUDRACT:2007-001716-23); full approval from United Kingdom Medicines and Healthcare products Regulatory Agency (MHRA) was granted. All participants gave written consent, and an adult with parental responsibility consented on behalf of their child for the offspring follow-up.

### Pregnancy phase

Individuals attending for early pregnancy (11–14 wk gestation) ultrasound scanning at 3 United Kingdom hospitals (University Hospital Southampton NHS Foundation Trust, Oxford University Hospitals NHS Foundation Trust, and Sheffield Hospitals NHS Trust) were invited to participate. Inclusion criteria were ≥18 y, singleton pregnancy, and gestational age <17 wk. Exclusion criteria were known metabolic bone disease, renal stones, hyperparathyroidism or hypercalciuria, taking medication known to interfere with fetal growth, fetal anomalies on ultrasonography, and individuals wishing to continue taking >400 IU/d vitamin D supplementation. A blood sample was collected, and serum 25(OH)D analyzed on the local hospital platform; those with a 25(OH)D between 25 and 100 nmol/l were eligible to enroll in the study.

Participants were randomly assigned 1:1 to either oral cholecalciferol 1000 IU/d or placebo from 14 to 17 wk gestation until delivery, as detailed previously [13]. All participants received standard antenatal care delivered by health professionals blinded to the study allocation. Participants could continue taking ≤400 IU/d vitamin D supplementation.

Assessments of lifestyle, health, and nutrition by interviewer-led questionnaire and anthropometry were performed at randomization and 34 wk gestation. Participants were asked to self-report their ethnicity from the following categories: White, Black Caribbean, Black African, Black Other, Indian, Pakistani, Bangladeshi, Chinese, Other Asian, or Other as specified by the participant. Blood samples were also collected at these study visits. Serum was stored at −80°C. 25(OH)D concentration was assessed by chemiluminescence immunoassay (Liaison automated platform, Diasorin). All samples were analyzed in a single batch at Medical Research Council (MRC) Human Nutrition Research. Within- and between-assay coefficients of variation were 4.1 and 6.1%, respectively.

### Offspring follow-up

Gestational age and birthweight were collected by a research nurse from participants’ medical records. Children born to participants recruited in Southampton were eligible to continue in the offspring follow-up. The duration of consumption of human milk was established in an interviewer-led questionnaire during a home-visit at 1 y of age. At ages 4 and 6–7 y, milk intake, use of vitamin D supplementation, physical activity, and medical diagnoses were established by an interviewer-administered questionnaire. Standing height was measured using a portable stadiometer (Leicester height measurer, Seca Ltd), to the nearest 0.1 cm. Weight was measured in light clothing using calibrated electronic scales (Seca Ltd) to the nearest 0.1 kg. Height, weight, and BMI z-scores for age and sex were calculated using British reference data [16,17].

Whole body and lumbar spine dual-energy X-ray absorptiometry (DXA) scans were obtained using a Hologic Discovery instrument (Hologic Inc.) in pediatric scan mode within 2 wk of birth and at ages 4 and 6–7 y. Outcomes of interest were bone area (BA), BMC, BMD, bone mineral apparent density (BMAD) [18], fat, and lean mass. Two researchers masked to treatment allocation reviewed the scans and those with substantial movement artifact affecting the whole body and/or both legs/both arms were excluded. In scans with movement artifact in 1 limb, the region of interest (ROI) of the unaffected limb was transposed into the limb with movement artifact. The DXA instrument underwent daily calibration using a spine phantom. The experimental coefficient of variation for this instrument when a spine phantom was repeatedly scanned in the same position 16 times, in a single session with no repositioning, was 0.68%.

All participants, children, and researchers remain blinded to the treatment allocation.

### Statistical analysis

The primary analysis was limited to children born at term (>37^+0^ wk^+d^ gestation) as these children had received full exposure to the study intervention. In a further sensitivity analysis, all children were included, irrespective of their gestation at birth.

Between-group comparisons on the effects of gestational vitamin D supplementation (maternal 25(OH)D, offspring outcomes) and comparing maternal characteristics for those included compared with not included in this follow-up were performed using *t* tests, Mann–Whitney U tests, and χ^2^ tests for normally distributed continuous, non-normally distributed continuous, and categorical variables, respectively. Results are presented as mean (SD), median (IQR) and *n* (%), respectively.

At ages 4 and 6–7 y, WBLH scans were used for the primary analysis [19]; at birth, whole body scans were used as isolating the skull ROI is not possible at this age. In secondary analysis, whole body scans were used to assess whether the use of WBLH DXA accounted for the different findings at birth and ages 6–7 y.

Although DXA outcomes were normally distributed, these were transformed to an SD scale using Fisher–Yates normal scores for the ease of comparison of effect sizes in regression models. Offspring sex and age at DXA were included in the models to increase the precision of the effect size estimates [20]. Height and weight were included to minimize the effect of bone size on BMD measured by DXA [21]. In a further model, the duration of the consumption of human breast milk and the use of vitamin D supplementation at ages 6–7 y were included as these differed between the 2 groups and may be associated with BMD in childhood [22,23]. Assumptions of normality and homoscedasticity of residuals were assessed after fitting linear regression models. The adjustment for multiple comparisons was not undertaken as the DXA outcomes are associated with each other (Supplemental Table 1) and applying formal adjustments to account for multiple comparisons can sabotage the interpretation of findings when the outcomes are associated [24]. While this is statistically appropriate, the post-hoc exploratory nature of the analysis of course provides less robust statistical evidence than would findings from a prespecified primary analysis.

We assessed for an interaction between the intervention and *1*) child’s sex and *2*) maternal 25(OH)D at randomization (using a threshold of 50 nmol/l).

Additionally, we examined the differences in the effect of pregnancy vitamin D supplementation in the children with scans at ages 4 and 6–7 y and at all 3 follow-up phases. Sex, age, height, and weight at DXA were included in these models, and in a further model, the duration of human milk consumption and the use of vitamin D supplements were additionally included only for the outcomes at ages 4 and 6–7 y.

All analysis was performed using Stata V17.0 (StataCorp LP).

## Results

**FIGURE 1:**
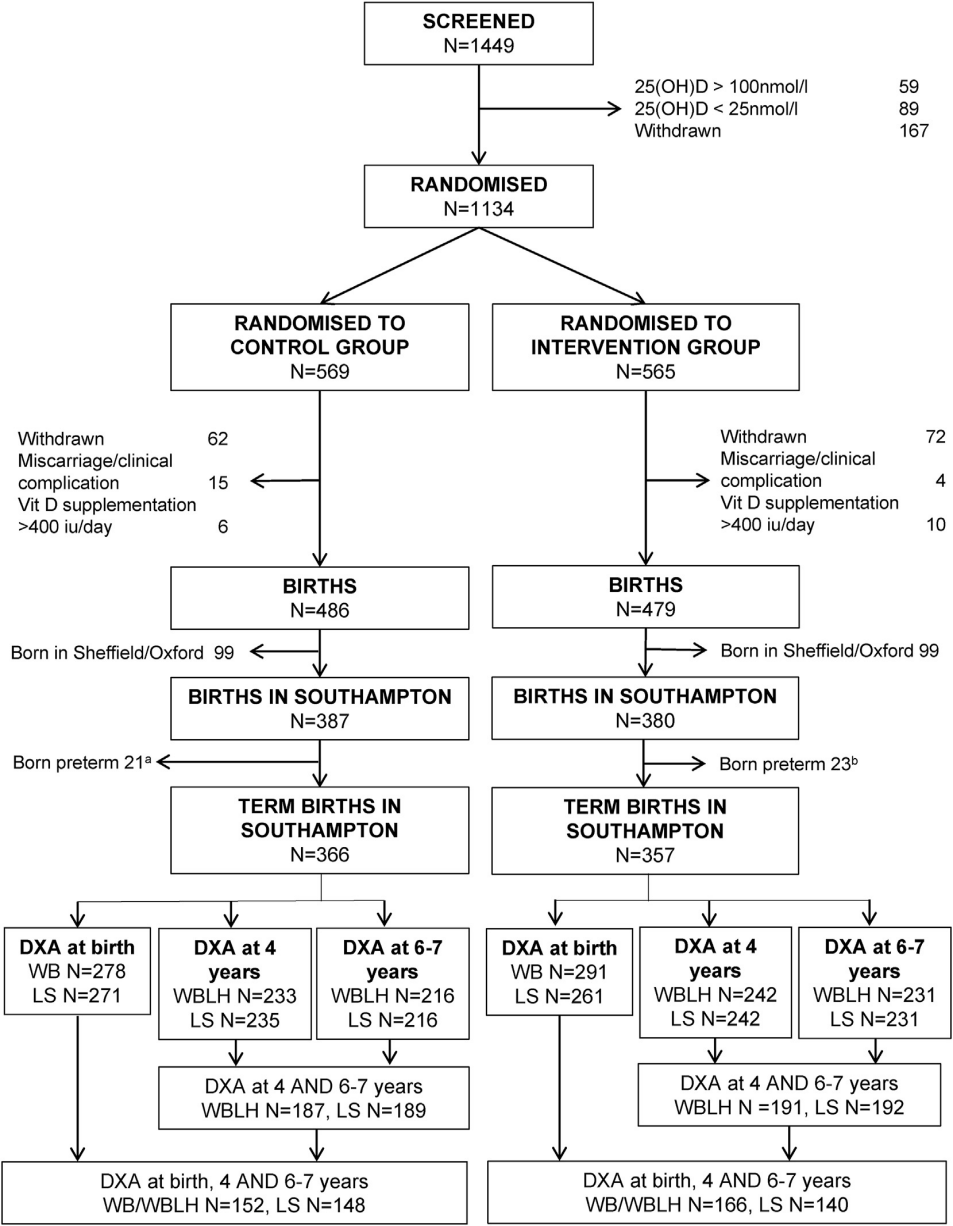
Participant flow diagram. DXA, dual-energy X-ray absorptiometry; LS, lumbar spine; WB, whole body; WBLH, whole-body-less-head. (a) Offspring born preterm in Southampton in the control group. DXA at birth—WB: *n =* 15 and LS: *n =* 12; 4 y—WBLH: *N =* 12 and LS: *N =* 12; 6–7 y—WBLH: *N =* 13 and LS: *N =* 13; (b) Offspring born preterm in Southampton in the intervention group. DXA at birth—WB: *n =* 12 and LS *n =* 9; 4 y—WBLH: *N =* 6 and LS: *N =* 6; and 6–7 y—WBLH: *N =* 9 and LS: *N =* 10.

Between 10 October, 2008, and 11 February, 2014, 1134 individuals agreed to participate in the original trial. A total of 965 continued in the study until delivery, of which 767 were born in Southampton (Figure 1). A total of 723 of these infants were born at term and 477 had a usable DXA scan (either at the whole body or spine) at age 4 y between 4 April, 2013, and 25 October, 2018. Between 22 November, 2016, and 12 April, 2022, 454 (63% of eligible children) attended the 6–7 y visit, of whom 447 had a usable DXA scan (Figure 1). Five children (1.1% of attendees; 2 placebo and 3 cholecalciferol) were aged between 8.0 and 8.1 y because of the delays in attendance resulting from the COVID-19 pandemic.

Maternal characteristics for the children attending at ages 6–7 y were similar between the randomly assigned groups (Table 1). Compared with those not participating in the follow-up, children who attended this visit were born to participants who were older, less likely to smoke in pregnancy, and had achieved a higher educational level (Supplemental Table 2). The children in the 2 groups were similar in age, sex, height, weight, and BMI z-score at the 6–7-y visit (Table 2). Children in the cholecalciferol group, on average, consumed human breast milk for a longer duration and a somewhat greater proportion were taking vitamin D supplements at 6–7 y (Table 2). Medical diagnoses were similar for each group (Supplemental Table 3).

**TABLE 1:** Characteristics of the mothers whose children had DXA data at 6–7 y.

	Placebo (*n =* 216)		Cholecalciferol (*n =* 231)	
	n		n	
Age at randomization, mean (SD) (y)	201	31.3 (4.8)	221	31.5 (4.7)
Height, mean (SD) (cm)	198	166.4 (6.4)	221	165.5 (6.3)
Weight, mean (SD) (kg)	201	73.8 (13.6)	221	71.8 (14.1)
BMI, median (IQR) (kg/m^2)^	198	25.7 (23.1, 29.6)	221	25.0 (22.4, 28.5)
Smoking in pregnancy, *n* (%)	183	10 (5.5)	203	12 (5.9)
White ethnicity, *n* (%)	201	197 (98.0)	219	211 (96.4)
Nulliparous, *n* (%)	200	85 (42.5)	221	92 (41.6)
Educated to degree level or higher, *n* (%)	199	162 (81.4)	219	184 (84.0)
25(OH)D in early pregnancy, mean (SD) (nmol/l)	211	45.0 (15.9)	228	46.3 (16.8)
25(OH)D in late pregnancy, mean (SD) (nmol/l)	196	43.4 (21.5)	216	68.1 (18.7)

Abbreviations: 25(OH)D, 25-hydroxyvitamin D; DXA, dual-energy X-ray absorptiometry.

**TABLE 2:** Anthropometry, bone densitometry, and body composition at ages 6–7 y by maternal randomization to placebo or 1000 IU/d cholecalciferol.

	Placebo		Cholecalciferol		*P*∗
	n		n		
Age, mean (range, SD) (y)	216	7.0 (range 6.1–8.1, SD 0.4)	231	7.1 (range 6.2–8.2, SD 0.5)	0.64
Male sex, *n* (%)	216	104 (48.2)	231	129 (55.8)	0.10
Birthweight, mean (SD) (g)	216	3592 (452)	231	3586 (468)	0.88
Gestation at birth, median (IQR) (wk)	216	40.4 (39.6, 41.1)	231	40.4 (39.6, 41.1)	0.99
Duration of breast feeding, median (IQR) (mo)	190	4 (0, 9)	210	6 (1, 11)	0.01
Use of vitamin D supplementation, *n* (%)	210	79 (37.6)	223	103 (46.2)	0.07
Milk intake, median (IQR) (pints/d)	212	0.5 (0.26,0.73)	225	0.5 (0.3, 0.7)	0.99
Physical activity, median (IQR) (min/wk)	179	30 (9, 60)	202	30 (0, 60)	0.61
Height, mean (SD) (cm)	210	123.6 (5.8)	224	123.8 (5.8)	0.68
Height z-score, mean (SD)	210	0.44 (1.04)	224	0.45 (1.04)	0.88
Weight, mean (SD) (kg)	210	24.7 (4.4)	224	24.7 (4.2)	0.94
Weight z-score, mean (SD)	210	0.35 (1.05)	224	0.35 (1.01)	0.95
BMI, mean (SD) (kg/m^2^)	210	16.1 (2.0)	224	16.0 (1.8)	0.80
BMI z-score, mean (SD)	210	0.13 (1.09)	224	0.12 (0.98)	0.90
Whole-body-less-head					
BA, mean (SD) (cm^2)^	216	949.23 (61.18)	231	954.41 (65.92)	0.39
BMC, mean (SD) (g)	216	558.60 (78.92)	231	570.40 (76.84)	0.11
BMD mean (SD) (g/cm^2^)	216	0.586 (0.053)	231	0.596 (0.048)	0.05
BMAD, mean (SD) (g/cm^3^)	216	0.0190 (0.0014)	231	0.0193 (0.0013)	0.04
Lean mass, mean (SD) (g)	216	14,255 (2257)	230	14,515 (2154)	0.21
Fat mass, median (IQR) (g)	216	5931 (4938, 7536)	230	5830 (4819, 7360)	0.39
Lumbar spine					
BA, mean (SD) (cm^2^)	216	30.06 (4.66)	231	30.14 (4.31)	0.85
BMC, mean (SD) (g)	215	19.51 (3.89)	230	19.84 (3.68)	0.37
BMD, mean (SD) (g/cm^2^)	215	0.647 (0.057)	230	0.656 (0.059)	0.12
BMAD, mean (SD) (g/cm^3^)	214	0.254 (0.028)	231	0.258 (0.029)	0.10

Abbreviations: BA, bone area; BMAD, bone mineral apparent density; BMC, bone mineral content; BMD, bone mineral density.

∗ *P* was obtained from *t* test, Wilcoxon rank sum test or *χ*^2^ test for normally distributed variables [displayed as mean (SD)], non-normally distributed variables [(displayed as median (interquartile range)], and categorical variables [(displayed as *n* (%)], respectively.

WBLH BMD and BMAD were greater in the cholecalciferol group than the placebo group at ages 6–7 y (Table 2). WBLH BA, BMC, and lean mass were also numerically greater in the cholecalciferol group, but this difference was not of statistical significance (Table 2). There was less evidence of an effect on lumbar spine parameters.

**FIGURE 2:**
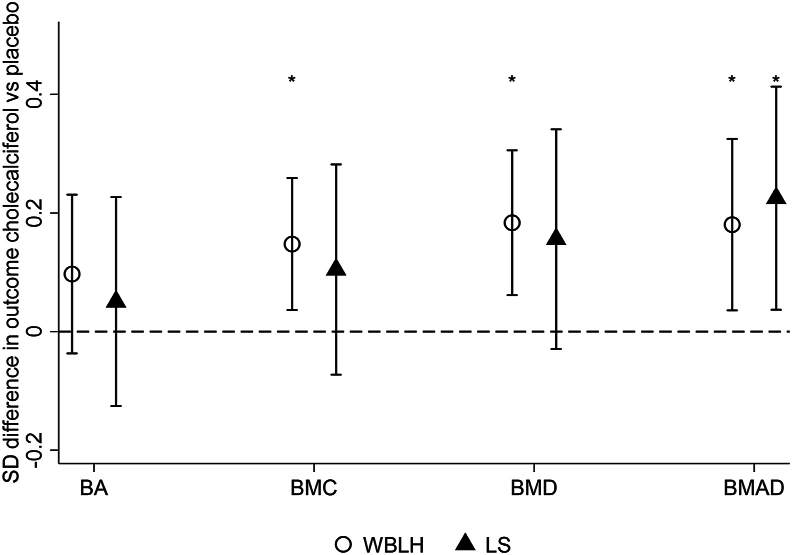
The effect of maternal pregnancy cholecalciferol supplementation compared with placebo on offspring WBLH (*n =* 384) and LS (*n =* 384 for BA and 382 for other outcomes) BA, BMC, BMD, and BMAD at ages 6–7 y. The point estimate shows the beta coefficient (95% CI) for the cholecalciferol group compared with placebo (effectively the mean difference in the measure between the 2 groups). A CI that does not cross *y* = 0 demonstrates a statistically significant (*P* < 0.05) difference between the 2 randomly assigned groups. Beta coefficients for standardized variables have been generated using linear regression and including adjustment for age at DXA, sex, height, weight, use of vitamin D supplementation at ages 6–7 y, and the duration of consumption of human milk. ∗ *P* < 0.05. BA, bone area; BMAD, bone mineral apparent density; BMC, bone mineral content; BMD, bone mineral density; CI, confidence interval; DXA, dual-energy X-ray absorptiometry; LS, lumbar spine; WBLH, whole-body-less-head.

Figure 2 shows the effect of gestational cholecalciferol supplementation compared with placebo on offspring bone outcomes with adjustment for age, sex, height, weight, duration of human milk consumption, and use of vitamin D supplementation at ages 6–7 y. This displays the positive effect of gestational cholecalciferol on WBLH BMC [0.15 SD, 95% confidence interval (CI): 0.04, 0.26], BMD (0.18 SD, 95% CI: 0.06, 0.31) and BMAD (0.18 SD, 95% CI: 0.04, 0.32), with similar direction of effects at the lumbar spine (data shown in Supplemental Tables 4 and 5). This model included 384 children (201 cholecalciferol and 183 placebo) because of missing covariates (47 duration of human milk consumption, 14 vitamin D supplementation at ages 6–7 y, 13 height/weight). In the fully adjusted model, WBLH lean mass was also greater in the cholecalciferol group (0.09 SD, 95% CI: 0.00, 0.17, *P =* 0.05). The findings were unchanged when whole body rather than WBLH scans were used (Supplemental Tables 4 and 5).

No significant statistical interaction between randomization and either *1*) child’s sex or *2*) maternal 25(OH)D at randomization with any of the WBLH or lumbar spine DXA outcomes was present (*P >* 0.05 for all).

**FIGURE 3:**
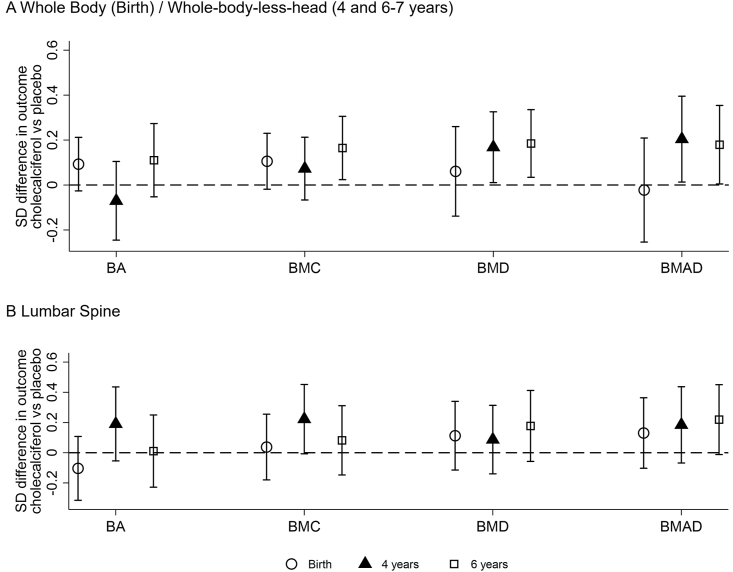
The effect of maternal pregnancy cholecalciferol supplementation compared with placebo on BA, BMC, BMD, and BMAD in children who had a DXA assessment at birth, 4, and 6–7 y for (A) whole body (birth)/whole-body-less head (4 and 6–7 y) (*n =* 263) and (B) lumbar spine (*n =* 236). Shown as beta (95% CI) for the cholecalciferol group compared with placebo. Beta coefficients for standardized variables have been generated using linear regression and including adjustment for age at DXA, sex, height (length at birth), weight at all ages, and additionally the use of vitamin D supplementation at time of DXA and the duration of consumption of human milk at ages 4 and 6–7 y. BA, bone area; BMAD, bone mineral apparent density; BMC, bone mineral content; BMD, bone mineral density; CI, confidence interval; DXA, dual-energy X-ray absorptiometry.

WBLH and lumbar spine DXA data were available at all of birth, 4 and 6–7 y for 263 and 236 children, respectively. In the analysis of this subset, with adjustment for age, sex, height (length at birth), and weight, no effect of gestational cholecalciferol on offspring WBLH bone outcomes at birth, but a positive effect of similar magnitude at ages 4 and 6–7 y (Figure 3, Supplemental Table 6). At the lumbar spine, there was a difference in BA and BMC noted at 4 y of age (although they did not reach statistical significance), which disappeared at 6–7 y, but a suggestion of greater lumbar spine BMAD at 6–7 y (Figure 3). Additional adjustment for vitamin D supplementation use in childhood and duration of consumption of human milk did not fundamentally change these findings (Supplemental Table 7).

An additional 24 children (13 placebo and 11 cholecalciferol) who had been born preterm (median 36.1 wk and range 32.3–36.9 wk) participated in the 6–7 y follow-up. The inclusion of these children in the analysis did not change the overall findings (Supplemental Table 8).

## Discussion

In this follow-up of the MAVIDOS randomized placebo-controlled trial, pregnancy supplementation with 1000 IU/d cholecalciferol increased offspring WBLH BMC and BMD at ages 6–7 y, with a similar direction of the effect at the lumbar spine. This strengthens the inference from previous MAVIDOS data, by showing persistence of the previously demonstrated positive effect of pregnancy vitamin D supplementation on offspring BMD at age 4 y [12].

These findings are consistent with those from the COPSAC_2010_ study in Denmark, in which 2800 IU/d compared with 400 IU/d cholecalciferol from mid-pregnancy until 1 wk after delivery resulted in higher whole body BMD and BMC adjusted for age, sex, height, and weight at age 6 y in 383 children, with similar effects for WBLH measurements [14]. The observed effect sizes (0.15–0.20 SD) in that study were of comparable magnitude to our results (0.15–0.18 SD). In contrast, O’Callaghan et al. [25] found no differences in WBLH BMD or BMC at 4 y of age in offsprings of children born to mothers randomly assigned to either placebo (*n =* 114), 4200 IU/wk (*n =* 126), 16,800 IU/wk (*n =* 120), or 28,000 IU/wk (*n =* 121) cholecalciferol in Bangladesh, but that trial was performed in a very different geographical location and population to the MAVIDOS and COPSAC_2010_ studies and the competing effects of other pre- and postnatal environmental factors, such as malnutrition, micronutrient and calcium deficiency, infections, and healthcare accessibility on skeletal development are important to consider when comparing the studies. Furthermore, it incorporated weekly supplementation, compared with the daily supplementation used in the 2 European studies; weekly supplementation may lead to greater fluctuations in maternal 25(OH)D status [26]. There are currently no intervention studies of pregnancy vitamin D supplementation with DXA assessment at an older age than the children in the MAVIDOS trial [2], but data from an Australian observational mother–offspring cohort study showed a positive association between maternal 25(OH)D at 18 wk gestation and offspring whole body BMD and BMC at 20 y of age [7].

The primary outcome of the MAVIDOS trial was offspring BMC at birth. There was no difference between the randomly assigned groups, although in stratified analyses, a positive effect of cholecalciferol supplementation on BMC in infants born in winter was observed [13]. It is therefore interesting that an effect across the whole cohort on BMD adjusted for age and sex of similar magnitude was observed at ages 4 y (0.17 SD, 95% CI: 0.00, 0.35) [12] and 6–7 y (0.16 SD, 95% CI: −0.01, 0.34). Notably, the analysis at 4 y included all children irrespective of gestation at birth, whereas in this analysis, we excluded a small number of children born preterm as they would have had less exposure to the pregnancy intervention and prematurity is recognized as a risk factor for low BMD in childhood [27]. However, sensitivity analysis including these children did not alter the overall findings. The adjustment for height attenuated the observed effect on BMD at 4 y of age, but not at 6–7 y. Given the recognized bias of greater body size on DXA measured BMD [18], this difference may reflect subtle differences in height of the 2 groups at the follow-up ages, with children in the cholecalciferol group being on average taller at age 4 y, but shorter at age 6–7 y. High-resolution peripheral quantitative computed tomography which has been undertaken on a subset of these children at ages 6–7 y, is less subject to influence by height and may provide further insight into the effect of gestational vitamin D supplementation on offspring bone microarchitecture and true volumetric BMD (analysis in progress).

Similarly to MAVIDOS, in the COPSAC_2010_ trial, there was no difference in offspring DXA outcomes at 3 y of age [28]. It is possible that this represents reduced statistical power in the COPSAC_2010_ given the smaller subset of children with successfully obtained DXA at age 3 y (*n =* 244) compared with 6 y (*n =* 383), although this is in contrast to MAVIDOS where DXA was available on more infants at birth than at ages 4 or 6–7 y. WBLH scans are the preferred site for DXA in childhood as the relatively large size and greater BMD of the skull can mask effects on the remainder of the skeleton [19,29]. However, at ages 6–7 y, the effect of the intervention on whole body outcomes were very similar to WBLH. It is therefore unlikely that this methodological difference in scan parameters at birth and 6–7 accounts for the differing effects at the 2 ages. Additionally, subgroup analysis of the children with DXA at all 3 timepoints, which were broadly similar to the whole cohort analysis, suggests that the changing effect is not because of the inclusion of different children at each age studied.

Our findings suggest that the effect of gestational cholecalciferol on offspring BMD may not result directly from increased calcium availability to the fetus as a difference in bone measures would have been expected in the neonatal period. We have previously reported in this trial that maternal supplementation resulted in an increase in umbilical cord blood 25(OH)D concentration, considered to reflect neonatal vitamin D status [30]. The circulating half-life of 25(OH)D is 2–3 wk [31]. Pregnancy vitamin D supplementation has been shown in 1 study in Bangladesh to improve infant 25(OH)D during the first 2 mo of life [32]; thus, the higher 25(OH)D at birth may allow for increased intestinal fractional calcium absorption during the first few months of postnatal life. Furthermore, while the vitamin D content of breast milk is low, risk factors associated with a lower breast milk antirachitic activity (the sum of vitamin D_2_, D_3_, 25(OH)D_2_, and 25(OH)D_3_) are similar to those for vitamin D deficiency (for example, lack of supplementation, season, and darker skin pigmentation) [33]. As such, improving maternal 25(OH)D status in the early postnatal period through pregnancy supplementation could be having an indirect effect on offspring bone development via increased breast milk vitamin D content [34]. Thus, mechanisms related to early postnatal vitamin D status might account for the evolution of an effect of pregnancy vitamin D supplementation on skeletal mineralization between birth and age 4 y, although previous studies of postnatal vitamin D supplementation in infancy have not shown an effect on BMD [25,35]. There are no reliable data on BMD between birth and age 3–4 y in trials of gestational vitamin D supplementation [2] to elucidate at what point an effect becomes apparent.

Alternatively, epigenetic mechanisms may be implicated in the evolving effect of pregnancy vitamin D on offspring skeletal development observed in our trial. Data from studies in both animals and humans, including intervention studies of gestational vitamin D supplementation [12,36], support a role for vitamin D status in epigenetic programming [37]. Indeed, in a small trial of gestational vitamin D supplementation in pregnancy [3800 IU (*n =* 3) compared with 400 IU (*n =* 7)], methylation differences in a number of genes, including those involved in bone and metabolic functions, were identified in offspring leucocytes [36]. Epigenetic mechanisms could underlie our previous observation that gestational vitamin D supplementation improves the anabolic response of the offspring’s bone to mechanical loading [15,38], which would explain the evolving effect of gestational vitamin D supplementation on the skeleton during childhood. Further replication of the epigenetic findings in larger studies is needed, alongside detailed biochemical studies to try to establish further potential mechanistic pathways.

There are no previous data relating maternal vitamin D status to offspring lumbar spine DXA measurements, but in an observational birth cohort study, Javaid et al. [5] reported no association between maternal 25(OH)D status in late pregnancy and offspring lumbar spine BA, but positive associations with lumbar spine BMC and BMD, at age 9 y. Overall, the effect of gestational cholecalciferol on offspring lumbar spine BMC and BMD at ages 6–7 y were weaker than for WBLH, but with a similar magnitude of effect for BMAD. Interestingly, the data on children with longitudinal DXA measurements suggests that the intervention resulted in greater lumbar spine BA and BMC at 4 y, but by 6–7 y, these parameters did not differ between the 2 randomly assigned groups, but BMAD (and to a lesser extent BMD) were greater in the children born to mothers randomly assigned to cholecalciferol. This suggests that early life vitamin D exposure may have an early positive effect on spinal growth coinciding with the period of rapid spinal growth in infancy [39], with a greater effect on spine mineralization from later in childhood. However, no effect of pregnancy vitamin D supplementation on offspring height was statistically apparent in our study or has been shown to persist beyond early infancy in other published trials [14,25,40].

The observed positive effect sizes are likely to be of clinical significance. Although increased physical activity in childhood may be associated with both greater BMD and higher fracture risk [41], on the whole, the evidence supports the notion that increasing BMD in childhood will reduce fracture risk [42]; in a study of over 6000 children, a 1 SD reduction in WBLH BMD at age 9 y was associated with a 1.12 increased odds of fracture over the subsequent 2 y [21]. The 0.18 SD difference in WBLH BMD between the 2 randomly assigned groups would therefore be expected to reduce offspring fracture risk, and indeed a lower fracture incidence was observed at age 6 y in post-hoc analysis of the COPSAC_2010_ trial [14]. Furthermore, while the reduction in odds of fracture in childhood may be small (∼2%), if this effect size on BMC and/or BMD were sustained into adult life, it would similarly be expected to translate to a clinically meaningful reduction in the burden of fracture in later life given the high frequency of fragility fracture in the population [43]. Further follow-up of this cohort of children during early adolescence is ongoing (commenced May 2023) to establish persistence of this effect and to obtain biological samples to undertake further work to elucidate mechanisms underlying the observed effects. Considering the low cost of pregnancy vitamin D supplementation, if these findings can be replicated and persist through puberty, increasing the currently recommended pregnancy supplementation guidance [[44], [45], [46]] to 1000 IU/d should be considered, particularly considering the other suggested benefits for maternal and offspring health [[47], [48], [49]].

The MAVIDOS study is the largest study of pregnancy vitamin D supplementation to assess offspring BMD and has the furthest duration of follow-up, but is not without limitations. Because of an ethical stipulation, only individuals with a baseline 25(OH)D between 25 and 100 nmol/l were eligible to take part in the trial. Thus, individuals who were very deficient in vitamin D and who would perhaps be expected to derive the greatest benefit from supplementation were excluded. This limitation would be expected to favor the null hypothesis; yet, despite this, a positive effect of vitamin D supplementation has been shown. However, replication of these findings in individuals with vitamin D deficiency is needed. The participants were predominately of White ethnicity, reflective of the local population, tended to be well-educated, and when considering the BMI distribution for both the mothers and offspring, overweight was common. This may limit the generalizability of our findings to other populations, and indeed, the differences between our findings and those of the study in Bangladesh [25] highlight that effects may differ depending on the presence of other risk factors for poor bone health such as poor nutrition. Only 47% of the original cohort participated in this follow-up phase, and this post-hoc exploratory follow-up was not included in the original trial design or statistical analysis plan. There were differences between the participants that continued in the study compared with those that did not, in that they tended to be born to mothers who were older, less likely to smoke, and more highly educated. This may introduce bias into the analysis and affect the generalizability of the study. While it would not be expected that allocation to the intervention or placebo (to which the participants remain blinded) would influence the likelihood of nonparticipation, the possibility of nonrandom dropout remains, with the associated potential to influence the results. Furthermore, because of missing covariates on the duration of human milk consumption and the use of vitamin D supplementation, the number of children included in the fully adjusted model reduced by 15% compared with the unadjusted model. Nonetheless, the effect size estimates were similar in the minimally and fully adjusted models.

In conclusion, we have demonstrated in a randomized placebo-controlled trial that supplementation with 1000 IU/d cholecalciferol from 14 to 17 wk pregnancy until delivery results in higher offspring BMD at ages 6–7 y. These findings suggest that pregnancy vitamin D supplementation may represent a population health strategy to improve bone health, although further work is needed to demonstrate persistence of this effect into adulthood, together with, ideally replication in additional studies.

## Author contributions

The authors’ responsibilities were as follows – RJM, EMC, KAW, IS, MKJ, NJB, KMG, CC, NCH, all members of the MAVIDOS study group: designed the research; RJM, EMC, IS: conducted research; SD, SRC: analyzed data or performed statistical analysis; RJM, SD& NCH: wrote the paper; NCH: had primary responsibility for the final content; all authors read and approved the final manuscript.

## Funding

This work was supported by Versus Arthritis United Kingdom (17702), Medical Research Council [MC_PC_21003; MC_PC_21001], Bupa Foundation, National Institute for Health Research (NIHR) Southampton Biomedical Research Centre, University of Southampton and University Hospital Southampton NHS Foundation Trust, and NIHR Biomedical Research Centre, University of Oxford. IS and AP were funded by the Medical Research Council (MRC) (programme code U105960371). RM and EMC are/were supported by NIHR Academic Clinical Lectureships. EMC was supported by a Wellcome Trust Clinical Research Fellowship. KMG is supported by the NIHR (NIHR Senior Investigator NF-SI-0515-10042) and Alzheimer’s Research United Kingdom (ARUK-PG2022A-008). The work leading to these results was supported by the European Union's Seventh Framework Programme (FP7/2007-2013), projects EarlyNutrition and ODIN under grant agreements numbers 289346 and 613977. We are extremely grateful to Merck GmbH for the kind provision of the Vigantoletten supplement. Merck GmbH had no role in the trial execution, data collection, analysis or manuscript preparation. The original protocol incorporated suggestions from the Arthritis Research United Kingdom Clinical Trials Collaboration. The funders had no other role in the study and the corresponding author had full access to all of the data and the final responsibility to submit for publication.

## Data availability

Data described in the manuscript, code book, and analytic code will be made available upon request pending application to and approval by the trial steering committee. Proposals should be directed to nch@mrc.soton.ac.uk. To gain access, data requestors will need to sign a data access agreement.

## Conflict of interest

RJM has received travel bursaries from Kyowa Kirin unrelated to this work. EMC has received travel bursaries or lecture fees from Eli Lilly, Pfizer, Thornton and Ross, and UCB, unrelated to this work. KMG has received reimbursement for speaking at conferences sponsored by companies selling nutritional products, and is part of an academic consortium that has received research funding from Abbott Nutrition, Nestec, BenevolentAI Bio Ltd., and Danone, outside the submitted work. MKJ reports consultancy and speaker fees from UCB, Amgen, and Kyowa Kirin. CC reports personal fees from ABBH, Amgen, Eli Lilly, GSK, Medtronic, Merck, Novartis, Pfizer, Roche, Servier, and Takeda, outside the submitted work. NCH reports personal fees, consultancy, lecture fees and honoraria from Alliance for Better Bone Health, AMGEN, MSD, Eli Lilly, Servier, Theramex, Shire, Consilient Healthcare, Kyowa Kirin, and Internis Pharma, outside the submitted work. KAW received Honoraria from Abbott Nutrition unrelated to this work. IS, SRC, and SD declare no conflicts of interest related to the submitted work.
