# Supplementary material for: Pregnancy vitamin D supplementation and offspring bone mineral density in childhood follow-up of a randomized controlled trial
Source: Am J Clin Nutr. 2024 Sep 19;120(5):1134–42. doi: 10.1016/j.ajcnut.2024.09.014 (PMC11600048; doi:10.1016/j.ajcnut.2024.09.014)
Supplement: Multimedia component 1 [file mmc1.docx]

**Pregnancy vitamin D and offspring bone mineral density in childhood**

**Follow-up of a randomised controlled trial**

**SUPPLEMENTARY TABLES**

Rebecca J Moon PhD^1,2^, Stefania D’ Angelo BSc^1,3^, Elizabeth M Curtis PhD^1^, Kate A Ward PhD^1^, Sarah R Crozier PhD^1,4^, Inez Schoenmakers PhD^5^, M.Kassim Javaid PhD^6,7^, Nicholas J Bishop MD^8^, Keith M Godfrey PhD^1,9^, Cyrus Cooper PhD^1,6,9^, Nicholas C Harvey PhD^1,9^ and the MAVIDOS Trial Group

**Supplementary Table 1:** Pairwise correlation between DXA outcomes at 6-7 years

**Supplementary Table 2**: Comparison of maternal characteristics for children that did and did not attend the 6–7-year follow-up

**Supplementary Table 3:** Parent-reported medical conditions in children attending the 6-7-year visit by randomization group

**Supplementary Table 4**: Effect of pregnancy cholecalciferol supplementation compared with placebo on offspring anthropometry and DXA outcomes at age 6-7 years

**Supplementary Table 5:** Effect of pregnancy cholecalciferol supplementation compared with placebo on offspring DXA outcomes at age 6-7 years

**Supplementary Table 6**: Effect of maternal pregnancy cholecalciferol supplementation compared to placebo on offspring DXA outcomes at birth, 4 and 6-7 years of age in children who had DXA assessment at all three ages.

**Supplementary Table 7:** Effect of maternal pregnancy cholecalciferol supplementation compared to placebo on offspring DXA outcomes at birth, 4 and 6-7 years of age in children who had DXA assessment at all three ages, with adjustment for all covariates.

**Supplementary Table 8:** Effect of pregnancy cholecalciferol supplementation compared with placebo on offspring anthropometry and DXA outcomes at age 6-7 years, including all children who attended the follow-up irrespective of their gestation at birth.

**Supplementary Table 1:** Pairwise correlation between DXA outcomes at 6-7 years

BA, bone area; BMC, bone mineral content; BMAD, bone mineral apparent density; BMD, bone mineral density; LS, lumbar spine; WBLH, whole body less head

p<0.001 for all correlations

|  | WBLH BA | WBLH BMC | WBLH BMD | WBLH BMAD | LS BA | LS BMC | LS BMD |
| --- | --- | --- | --- | --- | --- | --- | --- |
| WBLH BA | 1 |  |  |  |  |  |  |
| WBLH BMC | 0.878 | 1 |  |  |  |  |  |
| WBLH BMD | 0.637 | 0.926 | 1 |  |  |  |  |
| WBLH BMAD | 0.304 | 0.719 | 0.925 | 1 |  |  |  |
| LS BA | 0.363 | 0.428 | 0.409 | 0.328 | 1 |  |  |
| LS BMC | 0.498 | 0.620 | 0.614 | 0.519 | 0.896 | 1 |  |
| LS BMD | 0.476 | 0.634 | 0.652 | 0.580 | 0.275 | 0.669 | 1 |
| LS BMAD | 0.264 | 0.422 | 0.480 | 0.467 | 0.180 | 0.452 | 0.692 |

**Supplementary Table 2**: **Comparison of maternal characteristics for children that did and did not participate in the 6–7-year follow-up.**

|  | **Did not attend 6-7 year follow-up (n=276)** | | **Did attend 6-7 year follow-up (n=447)** | |
| --- | --- | --- | --- | --- |
|  | **n** |  | **n** |  |
| Age at randomization, years | 70 | 29.8 (5.5) | 422 | 31.4 (4.8) |
| Height, cm | 268 | 165.9 (6.6) | 419 | 165.9 (6.4) |
| Weight, kg | 270 | 71.6 (14.4) | 422 | 72.8 (13.9) |
| BMI, kg/m^2^ | 268 | 24.9 (22.3,29.0) | 419 | 25.3 (22.6,29.3) |
| Smoking in pregnancy* | 228 | 41 (18.0) | 386 | 22 (5.7) |
| White ethnicity* | 268 | 250 (93.3) | 420 | 408 (97.1) |
| Nulliparous* | 267 | 111 (41.6) | 421 | 177 (42.0) |
| Educated to degree level or higher* | 267 | 179 (67.0) | 418 | 346 (82.8) |
| 25(OH)D in early pregnancy (nmol/l) | 270 | 44.2 (16.0) | 439 | 45.7 (16.4) |
| 25(OH)D in late pregnancy (nmol/l) | 234 | 50.4 (24.1) | 412 | 56.3 (23.5) |

Shown as mean (SD), n (%)* or median (IQR)+

**Supplementary Table 3: Parent-reported medical conditions in children attending the 6-7 year visit by randomization group**

|  | Placebo, n (%) | Cholecalciferol, n (%) |
| --- | --- | --- |
| Asthma | 6 (2.8) | 9 (3.9) |
| Eczema/Dermatitis | 5 (2.3) | 8 (3.5) |
| Allergy(ies) | 8 (3.7) | 2 (0.9) |
| ADHD | - | 1 (0.4) |
| Autism | 1 (0.5) | - |
| Tic disorder | - | 1 (0.4) |
| Other mental health issues | 1 (0.5) | - |
| Type 1 diabetes | 1 (0.5) | - |
| Epilepsy | - | 1 (0.4) |
| Glue Ear | 7 (3.2) | 6 (2.6) |
| Hearing impairment | 2 (0.9) | 2 (0.9) |
| Cleft palate | - | 1 (0.4) |
| Ophthalmological conditions | 2 (0.9) | 5 (2.2) |
| Migraine | 1 (0.5) | 2 (0.9) |
| Developmental dysplasia of the hip | - | 1 (0.4) |
| Mastocystosis | 1 (0.5) | - |
| Nephrotic syndrome | - | 1 (0.4) |
| Recurrent urinary tract infection | 1 (0.5) | - |
| Chronic constipation | 2 (0.9) | 1 (0.4) |

**Supplementary Table 4**: **Effect of pregnancy cholecalciferol supplementation compared with placebo on offspring anthropometry and DXA outcomes at age 6-7 years**

Beta represents the SD difference between maternal cholecalciferol group compared to the placebo group.

|  | **Cholecalciferol vs placebo** | | | | | | | | | | | |
| --- | --- | --- | --- | --- | --- | --- | --- | --- | --- | --- | --- | --- |
|  | **Unadjusted** | | | **Adjusted for age, sex** | | | **Adjusted for age, sex, and height** | | | **Adjusted for age, sex, and weight** | | |
|  | **N** | **β (95% CI)** | **p** | **N** | **β (95% CI)** | **p** | **N** | **β (95% CI)** | **p** | **N** | **β (95% CI)** | **p** |
| Weight z-score | 434 | -0.004 (-0.19,0.18) | 0.96 | 434 | -0.01 (-0.20, 0.17) | 0.88 |  |  |  |  |  |  |
| Height z-score | 434 | 0.02 (-0.17,0.20) | 0.86 | 434 | 0.01 (-0.18, 0.20) | 0.93 |  |  |  |  |  |  |
| BMI z-score | 434 | 0.004 (-0.18,0.19) | 0.96 | 434 | -0.00 (-0.19,0.19) | 0.99 |  |  |  |  |  |  |
| ***Whole body less head*** | | | | | | | | | |  |  |  |
| BA | 447 | 0.08 (-0.11,0.26) | 0.40 | 447 | 0.08 (-0.10, 0.25) | 0.38 | 434 | 0.08 (-0.04,0.20) | 0.21 | 434 | 0.09 (-0.07,0.24) | 0.27 |
| BMC | 447 | 0.15 (-0.03,0.33) | 0.10 | 447 | 0.13 (-0.04, 0.30) | 0.13 | 434 | 0.12 (0.01,0.23) | 0.03 | 434 | 0.13 (0.01,0.25) | 0.03 |
| BMD | 447 | 0.20 (0.02,0.38) | 0.03 | 447 | 0.16 (-0.01, 0.34) | 0.06 | 434 | 0.15 (0.02,0.29) | 0.02 | 434 | 0.16 (0.05,0.28) | 0.006 |
| BMAD | 447 | 0.21 (0.03,0.39) | 0.03 | 447 | 0.16 (-0.01, 0.34) | 0.06 | 434 | 0.15 (-0.005,0.31) | 0.06 | 434 | 0.16 (0.03,0.29) | 0.02 |
| Lean | 446 | 0.13 (-0.06,0.31) | 0.17 | 446 | 0.08 (-0.09,0.24) | 0.38 | 433 | 0.07 (-0.03,0.17) | 0.18 | 433 | 0.08 (-0.02,0.17) | 0.10 |
| Fat | 447 | -0.11 (-0.29,0.07) | 0.25 | 446 | -0.07 (-0.25, 0.10) | 0.41 | 433 | -0.08 (-0.24,0.07) | 0.28 | 433 | -0.08 (-0.17,0.02) | 0.12 |
| ***Whole body*** |  |  |  |  |  |  |  |  |  |  |  |  |
| BA | 447 | 0.12 (-0.06,0.31) | 0.18 | 447 | 0.11 (-0.07,0.29) | 0.22 | 434 | 0.11 (-0.02,0.23) | 0.10 | 434 | 0.11 (-0.04,0.27) | 0.16 |
| BMC | 447 | 0.17 (-0.01,0.35) | 0.06 | 447 | 0.15 (-0.03,0.33) | 0.11 | 434 | 0.14 (0.01,0.28) | 0.04 | 434 | 0.15 (0.008,0.30) | 0.04 |
| BMD | 447 | 0.19 (0.002,0.37) | 0.05 | 447 | 0.15 (-0.03,0.33) | 0.10 | 434 | 0.16 (-0.004,0.32) | 0.06 | 434 | 0.17 (0.01,0.32) | 0.03 |
| BMAD | 447 | 0.19 (0.01,0.38) | 0.04 | 447 | 0.16 (-0.02,0.34) | 0.08 | 434 | 0.17 (-0.01,0.34) | 0.06 | 434 | 0.17 (0.01,0.34) | 0.04 |
| Lean | 446 | 0.13 (-0.05,0.31) | 0.16 | 446 | 0.08 (-0.09,0.24) | 0.37 | 433 | 0.07 (-0.03,0.17) | 0.18 | 433 | 0.08 (-0.01,0.17) | 0.09 |
| Fat | 446 | -0.10 (-0.28,0.08) | 0.29 | 446 | -0.07 (-0.24,0.11) | 0.44 | 433 | -0.08 (-0.23,0.08) | 0.32 | 433 | -0.07 (-0.16,0.03) | 0.15 |
| ***Lumbar spine*** | | | | | | | | | |  |  |  |
| BA | 447 | 0.02 (-0.16,0.20) | 0.83 | 447 | -0.01 (-0.19, 0.17) | 0.89 | 434 | 0.0002 (-0.17,0.17) | 0.99 | 434 | 0.003 (-0.18,0.18) | 0.97 |
| BMC | 445 | 0.08 (-0.10,0.27) | 0.37 | 445 | 0.07 (-0.12,0.25) | 0.49 | 432 | 0.07 (-0.10,0.23) | 0.43 | 432 | 0.07 (-0.10,0.25) | 0.42 |
| BMD | 445 | 0.15 (-0.04,0.33) | 0.12 | 445 | 0.16 (-0.02,0.34) | 0.09 | 432 | 0.15 (-0.03,0.33) | 0.10 | 432 | 0.15 (-0.02,0.33) | 0.08 |
| BMAD | 445 | 0.15 (-0.03,0.34) | 0.11 | 445 | 0.19 (0.01,0.37) | 0.04 | 432 | 0.19 (0.005,0.37) | 0.04 | 432 | 0.19 (0.01,0.37) | 0.04 |

|  | **Cholecalciferol vs placebo** | | | | | | | | |
| --- | --- | --- | --- | --- | --- | --- | --- | --- | --- |
|  | **Adjusted for age, sex, height, breastfeeding, and D supplements** | | | **Adjusted for age, sex, weight, breastfeeding, and D supplements** | | | **Adjusted for age, sex, vitamin D supplement use at age 6-7, duration of breastfeeding, height, and weight** | | |
|  | **N** | **β (95% CI)** | **N** | **N** | **β (95% CI)** | **p** | **N** | **β (95% CI)** | **p** |
| ***Whole body less head*** | | | | | | | | | |
| BA | 384 | 0.10 (-0.04,0.23) | 0.15 | 384 | 0.15 (-0.02,0.31) | 0.08 | 384 | 0.10 (-0.04,0.23) | 0.15 |
| BMC | 384 | 0.13 (0.007,0.25) | 0.04 | 384 | 0.18 (0.05,0.31) | 0.005 | 384 | 0.15 (0.04,0.26) | 0.009 |
| BMD | 384 | 0.15 (0.007,0.29) | 0.04 | 384 | 0.20 (0.08,0.33) | 0.002 | 384 | 0.18 (0.06,0.31) | 0.003 |
| BMAD | 384 | 0.14 (-0.03,0.31) | 0.11 | 384 | 0.18 (0.03,0.32) | 0.02 | 384 | 0.18 (0.04,0.32) | 0.02 |
| Lean | 383 | 0.06 (-0.05,0.17) | 0.31 | 383 | 0.11 (0.02,0.21) | 0.03 | 383 | 0.09 (0.00,0.17) | 0.05 |
| Fat | 383 | -0.14 (-0.30,0.02) | 0.08 | 383 | -0.10 (-0.20,-0.003) | 0.04 | 383 | -0.09 (-0.19,0.01) | 0.07 |
| ***Whole body*** |  |  |  |  |  |  |  |  |  |
| BA | 384 | 0.12 (-0.01,0.26) | 0.08 | 384 | 0.18 (0.01,0.35) | 0.04 | 384 | 0.12 (-0.01,0.26) | 0.08 |
| BMC | 384 | 0.15 (-0.002,0.30) | 0.05 | 384 | 0.20 (0.04,0.35) | 0.01 | 384 | 0.17 (0.02,0.32) | 0.02 |
| BMD | 384 | 0.15 (-0.02,0.33) | 0.08 | 384 | 0.19 (0.03,0.36) | 0.02 | 384 | 0.18 (0.02,0.35) | 0.03 |
| BMAD | 384 | 0.15 (-0.04,0.34) | 0.12 | 384 | 0.17 (-0.003,0.35) | 0.05 | 384 | 0.18 (0.01,0.36) | 0.04 |
| Lean | 383 | 0.06 (-0.05,0.17) | 0.32 | 383 | 0.11 (0.01,0.20) | 0.02 | 383 | 0.09 (0.003,0.17) | 0.04 |
| Fat | 383 | -0.13 (-0.29,0.03) | 0.10 | 383 | -0.09 (-0.19,0.004) | 0.06 | 383 | -0.08 (-0.18,0.01) | 0.09 |
| ***Lumbar spine*** | | | | | | | | | |
| BA | 384 | 0.06 (-0.12,0.23) | 0.54 | 384 | 0.08 (-0.10,0.26) | 0.40 | 384 | 0.05 (-0.13,0.23) | 0.57 |
| BMC | 382 | 0.10 (-0.08,0.28) | 0.27 | 382 | 0.13 (-0.05,0.31) | 0.16 | 382 | 0.10 (-0.07,0.28) | 0.25 |
| BMD | 382 | 0.14 (-0.05,0.33) | 0.15 | 382 | 0.16 (-0.02,0.35) | 0.08 | 382 | 0.16 (-0.03,0.34) | 0.10 |
| BMAD | 382 | 0.20 (0.008,0.40) | 0.04 | 382 | 0.21 (0.02,0.40) | 0.03 | 382 | 0.22 (0.04,0.41) | 0.02 |

**Supplementary Table 5**: **Effect of pregnancy cholecalciferol supplementation compared with placebo on offspring DXA outcomes at age 6-7 years**

Beta represents the difference between maternal cholecalciferol group compared to the placebo group with outcomes in original units.

|  | **Cholecalciferol vs placebo** | | | | | | | | |
| --- | --- | --- | --- | --- | --- | --- | --- | --- | --- |
|  | **Unadjusted** | | | **Adjusted for age and sex** | | | **Adjusted for age, sex, and height** | | |
|  | **N** | **β (95% CI)** | **p** | **N** | **β (95% CI)** | **p** | **N** | **β (95% CI)** | **p** |
| ***Whole body less head*** | | | | | | | | | |
| BA (cm^2^) | 447 | 5.18 (-6.67,17.02) | 0.39 | 447 | 5.12 (-6.13,16.37) | 0.37 | 434 | 5.14 (-2.77,13.06) | 0.20 |
| BMC (g) | 447 | 11.80 (-2.68,26.25) | 0.11 | 447 | 10.03 (-3.56,23.62) | 0.15 | 434 | 9.67 (0.81,18.53) | 0.03 |
| BMD (g/cm^2^) | 447 | 0.01 (-0.0001,0.02) | 0.05 | 447 | 0.01 (-0.001,0.02) | 0.10 | 434 | 0.007 (0.003,0.01) | 0.04 |
| BMAD (g/cm^3^) | 447 | 0.0003 (0.000009,0.0005) | 0.04 | 447 | 0.0002 (-0.00004,0.0004) | 0.10 | 434 | 0.0002 (-0.00003,0.0004) | 0.09 |
| Lean (g) | 446 | 259.8 (-150.67,670.22) | 0.21 | 446 | 144.3 (-232.6,521.2) | 0.45 | 433 | 131.19 (-102.57,364.94) | 0.27 |
| Fat (g) | 446 | -242.91 (-706.25,220.43) | 0.30 | 446 | -161.0 (-601.5,279.6) | 0.47 | 433 | -191.46 (-589.76,206.85) | 0.35 |
| ***Whole body*** |  |  |  |  |  |  |  |  |  |
| BA (cm^2^) | 447 | 8.29 (-4.67,21.25) | 0.21 | 447 | 7.40 (-5.37,20.16) | 0.26 | 434 | 6.87 (-1.99,15.73) | 0.13 |
| BMC (g) | 447 | 19.22 (-0.29,38.74) | 0.05 | 447 | 16.33 (-2.95,35.61) | 0.10 | 434 | 16.36 (1.46,31.25) | 0.03 |
| BMD (g/cm^2^) | 447 | 0.01 (0.001,0.02) | 0.03 | 447 | 0.01 (-0.001,0.02) | 0.07 | 434 | 0.01 (0.0006,0.02) | 0.04 |
| BMAD (g/cm^3^) | 447 | 0.0003 (0.000009,0.0005) | 0.04 | 447 | 0.0002 (-0.00003,0.0005) | 0.09 | 434 | 0.0002 (-0.0001,0.0005) | 0.06 |
| Lean (g) | 446 | 289.69 (-148.74,728.11) | 0.20 | 446 | 162.1 (-239.8,565.0) | 0.43 | 433 | 146.03 (-100.35,392.41) | 0.25 |
| Fat (g) | 446 | -229.06 (-399.90,241.77) | 0.34 | 446 | -151.8 (-601.5,297.8) | 0.51 | 433 | -183.32 (-586.79,222.15) | 0.38 |
| ***Lumbar spine*** | | | | | | | | | |
| BA (cm^2^) | 447 | 0.08 (-0.75,0.91) | 0.85 | 447 | -0.07 (-0.89,0.75) | 0.86 | 434 | -0.007 (-0.78,0.77) | 0.99 |
| BMC (g) | 445 | 0.32 (-0.38,1.03) | 0.37 | 445 | 0.24 (-0.46,0.94) | 0.49 | 432 | 0.26 (-0.39,0.90) | 0.43 |
| BMD (g/cm^2^) | 445 | 0.009 (-0.002,0.02) | 0.12 | 445 | 0.01 (-0.001,0.02) | 0.09 | 432 | 0.009 (-0.002,0.02) | 0.10 |
| BMAD (g/cm^3^) | 445 | 0.004 (-0.0009,0.01) | 0.10 | 445 | 0.006 (0.0003,0.01) | 0.04 | 432 | 0.006 (0.0002,0.011) | 0.04 |

|  | **Cholecalciferol vs placebo** | | | | | | | | |
| --- | --- | --- | --- | --- | --- | --- | --- | --- | --- |
|  | **Adjusted for age, sex, and weight** | | | **Adjusted for age, sex, duration of human milk consumption, and D supplements, height** | | | **Adjusted for age, sex, vitamin D supplement use at age 6-7, duration of human milk consumption, weight** | | |
|  | **N** | **β (95% CI)** | **p** | **N** | **β (95% CI)** | **p** | **N** | **β (95% CI)** | **p** |
| ***Whole body less head*** | | | | | | | | | |
| BA (cm^2^) | 434 | 5.61 (-4.26,15.48) | 0.27 | 384 | 6.40 (-2.14,14.93) | 0.14 | 384 | 9.74 (-0.80,20.29) | 0.07 |
| BMC (g) | 434 | 10.45 (0.89,20.01) | 0.03 | 384 | 9.81 (0.16,19.47) | 0.05 | 384 | 14.27 (4.04,24.50) | 0.006 |
| BMD (g/cm^2^) | 434 | 0.01 (0.002,0.01) | 0.01 | 384 | 0.01 (-0.001,0.01) | 0.08 | 384 | 0.009 (0.003,0.02) | 0.005 |
| BMAD (g/cm^3^) | 434 | 0.0002 (0.00002,0.0004) | 0.03 | 384 | 0.0002 (-0.0001,0.0004) | 0.18 | 384 | 0.0002 (.00002,0.0004) | 0.03 |
| Lean (g) | 433 | 149.08 (-51.64,349.79) | 0.15 | 383 | 91.03 (-161.45,343.51) | 0.48 | 383 | 209.93 (-0.14,420.01) | 0.05 |
| Fat (g) | 433 | -170.34 (-380.11,39.44) | 0.11 | 383 | -311.11 (-718.64,96.42) | 0.13 | 383 | -219.39 (-439.64,0.86) | 0.05 |
| ***Whole body*** |  |  |  |  |  |  |  |  |  |
| BA (cm^2^) | 434 | 7.41 (-3.72,18.54) | 0.19 | 384 | 8.15 (-1.50,17.80) | 0.10 | 384 | 11.96 (-0.004,23.93) | 0.05 |
| BMC (g) | 434 | 17.28 (1.69,32.9) | 0.03 | 384 | 16.75 (0.52,32.99) | 0.04 | 384 | 22.08 (5.25,38.90) | 0.01 |
| BMD (g/cm^2^) | 434 | 0.01 (0.002,0.02) | 0.02 | 384 | 0.01 (-0.001,0.02) | 0.06 | 384 | 0.01 (0.002,0.02) | 0.02 |
| BMAD (g/cm^3^) | 434 | 0.0002 (0.00001,0.0005) | 0.04 | 384 | 0.0002 (-.00006,0.0005) | 0.12 | 384 | 0.0002 (−0.000004,0.0005) | 0.05 |
| Lean (g) | 433 | 165.38 (-40.66,371.42) | 0.12 | 383 | 93.80 (-172.30,359.90) | 0.49 | 383 | 221.6 (5.17,437.94) | 0.05 |
| Fat (g) | 433 | -160.62 (-370.25,49.02) | 0.13 | 383 | -303.72 (-717.89,110.44) | 0.15 | 383 | -208.6 (-428.7,11.6) | 0.06 |
| ***Lumbar spine*** | | | | | | | | | |
| BA (cm^2^) | 434 | 0.007 (-0.81,0.83) | 0.99 | 384 | 0.24 (-0.56,1.04) | 0.55 | 384 | 0.35 (-0.48,1.19) | 0.41 |
| BMC (g) | 432 | 0.28 (-0.40,0.96) | 0.42 | 382 | 0.38 (-0.30,1.06) | 0.28 | 382 | 0.50 (-0.21,1.21) | 0.17 |
| BMD (g/cm^2^) | 432 | 0.009 (-0.001,0.02) | 0.08 | 382 | 0.008 (-0.003,0.02) | 0.14 | 382 | 0.01 (-0.001,0.02) | 0.08 |
| BMAD (g/cm^3^) | 432 | 0.006 (0.0005,0.01) | 0.03 | 382 | 0.006 (0.0003,0.012) | 0.04 | 382 | 0.006 (0.001,0.01) | 0.03 |

|  | **Cholecalciferol vs placebo** | | |
| --- | --- | --- | --- |
|  | **Adjusted for age, sex, vitamin D supplement use at age 6-7, duration of human milk consumption, height and weight** | | |
|  | **N** | **β (95% CI)** | **p** |
| ***Whole body less head*** | | | |
| BA (cm^2^) | 384 | 6.37 (-2.18,14.92) | 0.14 |
| BMC (g) | 384 | 11.49 (2.60,20.38) | 0.01 |
| BMD (g/cm^2^) | 384 | 0.008 (0.002,0.01) | 0.008 |
| BMAD (g/cm^3^) | 384 | 0.0002 (0.00002,0.0004) | 0.03 |
| Lean (g) | 383 | 160.4 (-27.4,348.2) | 0.09 |
| Fat (g) | 383 | -163.9 (-357.2,29.3) | 0.10 |
| ***Whole body*** | | | |
| BA (cm^2^) | 384 | 8.11 (-1.56,17.79) | 0.10 |
| BMC (g) | 384 | 18.68 (3.02,34.34) | 0.02 |
| BMD (g/cm^2^) | 384 | 0.01 (0.002,0.02) | 0.02 |
| BMAD (g/cm^3^) | 384 | 0.0003 (0.00001,0.0005) | 0.04 |
| Lean (g) | 383 | 169.4 (-22.9,361.7) | 0.08 |
| Fat (g) | 383 | -153.6 (-347.2,40.1) | 0.12 |
| ***Lumbar spine*** | | | |
| BA (cm^2^) | 384 | 0.22 (-0.58,1.02) | 0.59 |
| BMC (g) | 382 | 0.40 (-0.29,1.08) | 0.25 |
| BMD (g/cm^2^) | 382 | 0.009 (-0.001,0.02) | 0.09 |
| BMAD (g/cm^3^) | 382 | 0.007 (0.001,0.01) | 0.02 |

**Supplementary Table 6: Effect of maternal pregnancy cholecalciferol supplementation compared to placebo on offspring DXA outcomes at birth, 4 and 6-7 years of age in children who had DXA assessment at all three ages.** Beta represents the SD difference between maternal cholecalciferol group compared to the placebo group, adjusted for sex and age, height (length at birth) and weight at DXA.

|  | **n** | **Birth** | | **4 years** | | | **6-7 years** | |
| --- | --- | --- | --- | --- | --- | --- | --- | --- |
|  |  | **β (95% CI)** | **p** | **β (95% CI)** | | **p** | **β (95% CI)** | **p** |
| ***Whole body (birth) / Whole-body-less-head (4 and 6-7 years)*** | | | | | |  |  |  |
| BA | 263 | 0.09 (-0.03,0.21) | 0.13 | | -0.07 (-0.25,0.10) | 0.43 | 0.11 (-0.05,0.27) | 0.18 |
| BMC | 263 | 0.11 (-0.02,0.23) | 0.10 | | 0.07 (-0.07,0.21) | 0.30 | 0.16 (0.02,0.30) | 0.02 |
| aBMD | 263 | 0.06 (-0.14,0.26) | 0.55 | | 0.17 (0.01,0.33) | 0.04 | 0.18 (0.03,0.34) | 0.02 |
| BMAD | 263 | -0.02 (-0.26,0.21) | 0.85 | | 0.20 (0.01,0.40) | 0.04 | 0.18 (0.005,0.35) | 0.04 |
| Lean | 263 | 0.08 (-0.06,0.22) | 0.25 | | 0.07 (-0.06,0.19) | 0.30 | 0.08 (-0.03,0.19) | 0.15 |
| Fat | 263 | -0.05 (-0.24,0.13) | 0.59 | | -0.07 (-0.21,0.08) | 0.37 | -0.06 (-0.18,0.05) | 0.30 |
| ***Lumbar spine*** | | | | | |  |  |  |
| BA | 236 | -0.10 (-0.31,0.11) | 0.34 | | 0.19 (-0.05,0.44) | 0.13 | 0.01 (-0.23,0.25) | 0.93 |
| BMC | 236 | 0.04 (-0.18,0.25) | 0.73 | | 0.22 (-0.01,0.45) | 0.06 | 0.08 (-0.15,0.31) | 0.48 |
| aBMD | 236 | 0.11 (-0.11,0.34) | 0.33 | | 0.09 (-0.14,0.31) | 0.45 | 0.18 (-0.06,0.41) | 0.14 |
| BMAD | 236 | 0.13 (-0.10,0.36) | 0.27 | | 0.18 (-0.07,0.44) | 0.15 | 0.22 (-0.01,0.45) | 0.06 |

**Supplementary Table 7: Effect of maternal pregnancy cholecalciferol supplementation compared to placebo on offspring DXA outcomes at birth, 4 and 6-7 years of age in children who had DXA assessment at all three ages with adjustment for covariates.** Beta represents the SD difference between maternal cholecalciferol group compared to the placebo group, adjusted for sex age at DXA, height, weight, duration of human milk consumption (only for outcomes at 4 and 6-7 years), and vitamin D supplement use (only for outcomes at 4 and 6-7 years).

|  | **Birth** | | | | **4 years** | | | **6-7 years** | | |
| --- | --- | --- | --- | --- | --- | --- | --- | --- | --- | --- |
|  | **n** | **β (95% CI)** | **p** | **n** | | **β (95% CI)** | **p** | **n** | **β (95% CI)** | **p** |
| ***Whole body (birth) / Whole-body-less-head (4 and 6-7 years)*** | | | | | | | | | | |
| BA | 234 | 0.06 (-0.07,0.18) | 0.37 | 234 | | -0.10 (-0.29,0.08) | 0.28 | 234 | 0.12 (-0.06,0.29) | 0.18 |
| BMC | 234 | 0.08 (-0.05,0.21) | 0.22 | 234 | | 0.08 (-0.07,0.23) | 0.28 | 234 | 0.19 (0.04,0.34) | 0.02 |
| aBMD | 234 | 0.05 (-0.16,0.27) | 0.63 | 234 | | 0.20 (0.03,0.36) | 0.02 | 234 | 0.22 (0.06,0.38) | 0.008 |
| BMAD | 234 | -0.01 (-0.26,0.23) | 0.91 | 234 | | 0.26 (0.06,0.46) | 0.01 | 234 | 0.22 (0.03,0.41) | 0.02 |
| Lean | 234 | 0.10 (-0.04,0.25) | 0.15 | 234 | | 0.08 (-0.05,0.22) | 0.22 | 234 | 0.08 (-0.04,0.20) | 0.19 |
| Fat | 234 | -0.07 (-0.27,0.13) | 0.48 | 232 | | -0.10 (-0.25,0.05) | 0.19 | 234 | -0.04 (-0.16,0.08) | 0.53 |
| ***Lumbar spine*** | | | | | | | | | | |
| BA | 207 | -0.09 (-0.32,0.13) | 0.42 | 207 | | 0.31 (0.05,0.57) | 0.02 | 207 | -0.03 (-0.28,0.23) | 0.84 |
| BMC | 207 | 0.04 (-0.19,0.28) | 0.72 | 207 | | 0.34 (0.10,0.59) | 0.007 | 207 | 0.05 (-0.20,0.30) | 0.71 |
| aBMD | 207 | 0.12 (-0.13,0.36) | 0.35 | 207 | | 0.14 (-0.10,0.37) | 0.25 | 207 | 0.18 (-0.07,0.44) | 0.16 |
| BMAD | 207 | 0.14 (-0.11,0.40) | 0.26 | 203 | | 0.30 (0.02,0.57) | 0.04 | 207 | 0.25 (-0.007,0.50) | 0.06 |

**Supplementary Table 8:** **Effect of pregnancy cholecalciferol supplementation compared with placebo on offspring anthropometry and DXA outcomes at age 6-7 years, including all children who attended the follow-up irrespective of their gestation at birth.**

Beta represents the SD difference between maternal cholecalciferol group compared to the placebo group.

|  | **Cholecalciferol vs placebo** | | | | | |
| --- | --- | --- | --- | --- | --- | --- |
|  | **Unadjusted** | | | **Adjusted for age, sex, weight, height, duration of human milk consumption, and D supplement use at 6-7 years** | | |
|  | **N** | **β (95% CI)** | **p** | **N** | **β (95% CI)** | **p** |
| Weight z-score | 477 | 0.01 (-0.17,0.19) | 0.91 | 411 | 0.02 (-0.04,0.08) | 0.60 |
| Height z-score | 477 | 0.03 (-0.15,0.20) | 0.77 | 411 | 0.02 (-0.03,0.07) | 0.38 |
| BMI z-score | 477 | 0.01 (-0.16,0.19) | 0.87 | 411 | 0.02 (-0.05,0.09) | 0.56 |
| ***Whole body less head*** |  |  |  |  |  |  |
| BA | 469 | 0.08 (-0.10,0.26) | 0.39 | 404 | 0.10 (-0.03,0.23) | 0.13 |
| BMC | 472 | 0.12 (-0.06,0.30) | 0.18 | 407 | 0.13 (0.02,0.24) | 0.02 |
| BMD | 469 | 0.18 (0.003,0.36) | 0.05 | 404 | 0.18 (0.06,0.30) | 0.003 |
| BMAD | 469 | 0.18 (0.003,0.36) | 0.05 | 404 | 0.17 (0.03,0.31) | 0.02 |
| Lean | 474 | 0.11 (-0.07,0.29) | 0.24 | 407 | 0.08 (-0.003,0.16) | 0.06 |
| Fat | 474 | -0.09 (-0.27,0.09) | 0.31 | 407 | -0.08 (-0.18,0.009) | 0.08 |
| ***Whole body*** |  |  |  |  |  |  |
| BA | 471 | 0.11 (-0.07,0.29) | 0.22 | 406 | 0.12 (-0.01,0.25) | 0.07 |
| BMC | 472 | 0.13 (-0.05,0.31) | 0.15 | 407 | 0.14 (-0.006,0.28) | 0.06 |
| BMD | 471 | 0.15 (-0.03,0.33) | 0.11 | 406 | 0.15 (-0.01,0.31) | 0.06 |
| BMAD | 471 | 0.15 (-0.03,0.33) | 0.11 | 406 | 0.15 (-0.02,0.32) | 0.09 |
| Lean | 474 | 0.11 (-0.07,0.29) | 0.22 | 407 | 0.08 (0.002,0.16) | 0.05 |
| Fat | 474 | -0.09 (-0.27,0.09) | 0.34 | 407 | -0.08 (-0.17,0.01) | 0.09 |
| ***Lumbar spine*** |  |  |  |  |  |  |
| BA | 472 | -0.02 (-0.20,0.16) | 0.80 | 407 | 0.007 (-0.16,0.18) | 0.93 |
| BMC | 470 | 0.05 (-0.13,0.23) | 0.57 | 405 | 0.08 (-0.09,0.25) | 0.37 |
| BMD | 470 | 0.15 (-0.03,0.33) | 0.10 | 405 | 0.17 (-0.006,0.35) | 0.06 |
| BMAD | 476 | 0.13 (-0.05,0.31) | 0.15 | 409 | 0.20 (0.02,0.38) | 0.03 |
